# Occupants’ Health and Their Living Conditions of Remote Indigenous Communities in New Zealand

**DOI:** 10.3390/ijerph17228340

**Published:** 2020-11-11

**Authors:** Bin Su, Lian Wu

**Affiliations:** 1 School of Architecture, Unitec Institute of Technology, 0600 Auckland, New Zealand; 2 School of Healthcare and Social Practice, Unitec Institute of Technology, 0600 Auckland, New Zealand; lwu@unitec.ac.nz

**Keywords:** dust mite, house, indoor allergen, indigenous community, indoor health, indoor microclimate, māori health, mould, living condition, respiratory health

## Abstract

The New Zealand Ministry of Health reported that respiratory disease affects 700,000 people, annually costs New Zealand NZ$7.05 billion, and is the third-highest cause of death. The hospitalisation rate for asthma of Māori communities is 2.0 higher than that of other ethnic groups, and hospitalisation rates for deprived homes are 2.3 times higher than those of the least deprived homes. Based on physical data and evidence, which were drawn from a mixed methodology that includes field studies of the indoor microclimate, dust-mite allergens, mould growth, and occupants’ Respiratory Health Survey of a number of sample houses of Māori communities in Minginui, Te Whaiti, Murupara, and Rotorua of New Zealand, the study identifies unhealthy indoor thermal conditions, thresholds or ranges of indoor micro-climate related to different levels of dust-mite allergen and mould growth, the most common type of indoor mould, and correlations between dust-mite and mould and correlations. The study not only identified that the poor health of occupants is closely related to their inadequate living conditions, but also identifies the threshold of indoor micro-climates to maintain indoor allergens at the acceptable level, which can be used as a guideline to maintain or improve indoor health conditions for future housing development or retrofitted old housing.

## 1. Introduction

Respiratory disease is one obvious consequence of inadequate housing [1,2,3], and the risk increases as indoor temperatures fall below 16 °C [3,4]. More than half of the people admitted to hospital with a poverty-related condition are there because of a respiratory problem such as asthma, bronchiolitis, acute infection or pneumonia [5]. According to the New Zealand Health Survey 2017/18, new respiratory disease affects 700,000 people, causes one in 10 hospital stays, costs New Zealand NZ$7 billion in healthcare every year, and is the third-highest cause of death. One in eight adults (12.5%) and one in seven children (14.3%) have asthma [6]. For all age groups, hospitalisation rates for asthma of Māori and Pasifika peoples are, respectively, 2.4 and 2.5 times higher than those of other ethnic groups, and hospitalisation rates for those in deprived homes are 2.3 times higher than those in the least deprived homes [5,6]. Respiratory diseases tend to be chronic in effect, are often developed in childhood and can shorten life expectancy [7].

The World Health Organisation recommends a minimum indoor temperature of 18 °C for houses, and 20–21 °C for more vulnerable occupants, such as older people and young children [8,9]. It is widely acknowledged that low-quality housing affects occupants’ health and wellbeing [1,8,10,11]. Previous studies show that the minimum threshold indoor temperature required for limiting respiratory infections is 16 °C [3,4]. Indoor temperatures below 12 °C can cause short-term increases in blood pressure and blood viscosity, which may increase winter morbidity and mortality due to heart attacks and strokes. When elderly people are exposed to indoor temperatures of 9 °C or below for two or more hours, their deep body temperature can start decreasing [12,13,14].

Most of the factors that adversely affect health, such as bacteria, viruses, fungi, mites, etc., have increases associated with high indoor relative humidity. Maintaining indoor relative humidity between 40% and 60% can minimise the indirect health effects [15]. New Zealand has a temperate climate with comfortable warm, dry summers and mild, wet winters. Housing thermal design not only focuses on winter indoor thermal comfort but also indoor health conditions related to high relative humidity. The abundance of two major causes of allergy and trigger asthma, mites and mould, in New Zealand housing increases proportionately with a rise in average indoor relative humidity. For a new house with sufficient insulation and double-glazed windows in Auckland, in order to keep the winter indoor mean relative humidity at 50% and within the range of 40–60%, the winter indoor mean air temperatures have to be heated up to and maintained at 21–22 °C by a central-heating system in a temperate climate with a mild and wet winter [16]. It is difficult and too expensive for houses only designed for temporary heating or without sufficient insulation to heat up the indoor air temperature of the whole house to 21–22 °C in order to maintain the indoor relative humidity below the thresholds of mould (60%) and mite (50%) survival and growth.

Both indoor relative humidity and temperature can impact indoor dust mite populations and allergen levels. Maintaining the indoor relative humidity below 50% can reduce dust mites and their allergens in the home; mite populations are almost eliminated in winter when indoor relative humidity is maintained within 40 to 50% [17,18,19]. A range of 60–80% for relative humidity provides ideal conditions for the reproduction of mites. The indoor relative humidity required by dust mites to thrive is 75–80% or higher, and dust mites prefer temperatures of around 18–25 °C. A decrease in indoor temperature (between 10 °C and 25 °C) can result in lower dust mite populations [17,20,21,22,23].

According to international and national standards, indoor relative humidity should be lower than 60% for optimum indoor air quality [24,25,26]. The threshold of indoor relative humidity for mould survival and growth conditions is 60%. Mould growth is likely on almost any building material if the equilibrium relative humidity of the material exceeds 75–80% [27,28,29]. Mould germination not only requires high relative humidity but also time (see Table 1) [30]. One option to prevent mould growth on indoor surfaces is to control the indoor relative humidity to a level below the threshold of mould germination [31,32].

**Figure 1:**
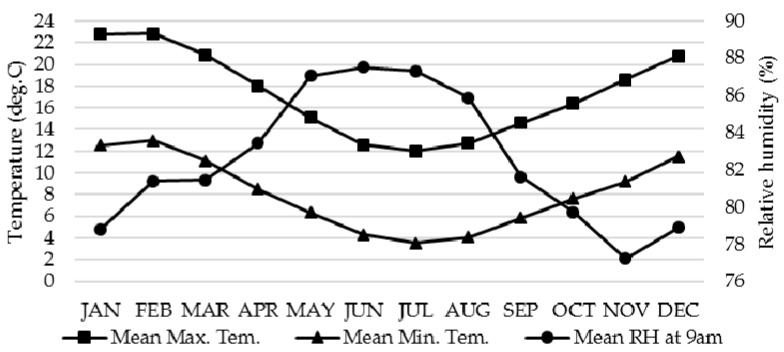
Climate data of Rotorua (Source: NIWA, the National Institute of Water and Atmospheric Research).

The living environment experienced by indigenous communities in modern countries is often substandard, with consequent implications for health and wellbeing. In total, 19 sample houses of Māori communities in Minginui, Te Whaiti, Murupara, and Rotorua are used for this study. ‘Māori’ is the modern umbrella term used to refer to the Polynesian peoples who established residence in New Zealand from about 1250 AD [33]. Sixteen sample houses (six houses in Minginui, five houses in Te Whaiti, and five houses in Murupara) without insulation or with limited old insulation were used for this study. Minginui, with 40 inhabited houses, is an old forestry town. Te Whaiti, with about 15 inhabited houses, is a village 8 km to the north of Minginui. Murupara is a small town with a population of 1650. The study area of Minginui, Te Whaiti, and Murupara is a remote and economically disadvantaged region, where the main industries are forestry and farming. As the three field-study sites are close to Rotorua, three sample houses with basic insulation of the Māori community in Rotorua were also used for this study. Rotorua has a temperate climate with mild and wet winters (see Figure 1).

The 16 sample houses with a light timber structure in Minginui, Te Whaiti, and Murupara were built in the 1920s to the 1970s. The floor areas of the 16 sample houses are 44 m^2^ to 342 m^2^. Roof materials are tin (eight houses), iron (five houses), aluminium (one house), tile (one house), and asbestos (one house). Wall materials are old weatherboard (14 houses), brick (one house), and concrete block (one house). Nine sample houses do not have any insulation in their envelopes; seven sample houses have only limited, old insulation in either their roof space only or both roof space and floor. For space heating, 12 sample houses used firewood as fuel for fireplaces, three sample houses used coal or firewood as fuel for stoves (cooking and space heating), one sample house did not use any space heating. Only one sample house used both a fireplace and an oil heater. Fifteen sample houses did not use any electricity as fuel for space heating. Three sample houses with the light timber structure in Rotorua were built in the 1980s to the 1990s with the basic insulation (Roof: 1.9, Wall: 1.5, Floor: 0.9) and single-glazed windows and used electronic heaters for space heating.

This study focuses on participants’ health conditions related to their living conditions (indoor thermal conditions and indoor allergens) of Māori communities in Minginui, Te Whaiti, Murupara and Rotorua of North Central Island of New Zealand. This study also provides guidelines or a strategy for new house development or retrofitting old house, with adequate space heating methods, to maintain indoor allergens at the acceptable level and to maintain healthy indoor conditions for occupants under the local climate with a mild and wet winter.

## 2. Methods

Field studies of the indoor microclimate of 19 sample houses (six houses in Minginui, five houses in Te Whaiti, five houses in Murupara and three houses in Rotorua) were carried out by the authors from March 2018 to January 2019. Air temperatures and relative humidity adjacent to floors and ceilings of different indoor spaces in the 19 sample houses and the shaded outdoor spaces under the eaves of the roofs were continuously measured and recorded at 15-min intervals, 24 h a day, by HOBO temperature and relative humidity (RH) loggers, from March 2018 to January 2019. All field-study data of the temperature and relative humidity of indoors and outdoors were converted into percentages of time in autumn and winter when indoor temperature and relative humidity were in different ranges for the purposes of identifying and comparing healthy indoor thermal conditions and identifying thresholds or ranges of indoor temperature and relative humidity related to mould germination, mould growth levels, the most common type of mould, and different levels of indoor dust-mite allergens. Field studies of dust-mite allergens and mould in 16 sample houses (13 sample houses in Minginui, Te Whaiti and Murupara; three sample houses in Rotorua) were carried out by the authors in the winter of 2018. According to the instructions for the Ventia™ Rapid Allergen Test, dust samples on the carpets of living rooms and bedrooms of the 16 sample houses were collected by a vacuum cleaner fitted with a DUSTREAM^®^ collector, and dust samples were then tested using the Rapid Test cassette. Test results can identify four different levels of dust-mite allergens: 1. Undetectable dust-mite allergens. 2. Low levels of dust-mite allergens (less than 0.2 micrograms per gram of dust). 3. Medium levels of dust-mite allergens (0.2–1.0 micrograms per gram of dust). 4. High levels of dust-mite allergens (≥1 microgram per gram of dust). For level 1 and 2, no action is needed to reduce the indoor mite-allergen level (acceptable levels), for level 3 and 4, action should be taken to reduce indoor dust-mite allergen levels to protect occupants’ health (unacceptable levels). Field-study data of indoor microclimate and dust-mite allergen test data can be used to identify thresholds and ranges of indoor temperature and relative humidity associated with different levels of indoor dust-mite allergens. The threshold of indoor temperature and relative humidity associated with the low level (the acceptable level) of indoor dust-mite allergens can be used as a guideline for housing thermal design and space heating to minimise indoor allergy problems.

According to the instructions of Biodet Services Ltd. (consulting industrial microbiologists), the researchers used clear, standard Sellotape to collect mould samples from the indoor surface areas of the 16 sample houses (13 sample houses in Minginui, Te Whaiti and Murupara; 3 sample houses in Rotorua). The Sellotape with the mould samples was then folded in non-stick baking paper and placed into a plastic bag; the samples were then sent to the local testing laboratory where they were examined both macroscopically and microscopically. Field-study data of indoor temperature and relative humidity can be used to estimate whether or not, or when, mould spores can germinate. Field-study data of indoor microclimate and mould tests can be used to identify thresholds and ranges of indoor temperature and relative humidity associated with different levels of mould growth. Field-study data of the indoor microclimate and mould and dust-mite allergen test data can be used to identify correlations between dust-mite allergens and mould growth

The respiratory survey questionnaire used in this study was adapted from the European Community Respiratory Health Survey, which has been used for 200,000 participants to date. The major change in the survey was that the survey questions were converted to an online form which is easy for researchers to use [34]. The questionnaire (total of 76 survey questions) investigates the participant’s basic health profile, respiratory symptom prevalence, risk factors, medication and related medical history, and all possible related factors. Research ethical approval (Application No. 2016–1007) was obtained from Unitec Research Ethics Committee (recognised by Health Research Council of New Zealand) before this study. After the occupants signed the consent form, the Respiratory Health Survey was carried out in 2018, and both Māori and English languages were used during the interviews and for explaining the questionnaire. A total of 23 occupants from 19 sample houses in Minginui, Te Whaiti, Murupara and Rotorua signed the consent form of the Respiratory Health Survey. In total, 20 participants from 15 sample houses in Minginui, Te Whaiti, Murupara and Rotorua finally took part in the Respiratory Health Survey. The Respiratory Health Survey results, field-study data of indoor temperature and relative humidity, and test data of dust mites and mould can be used to establish a relationship between occupants’ respiratory health conditions and indirect indoor health effects such as dust mites and mould.

## 3. Data Analysis

### 3.1. Indoor Thermal Conditions

#### 3.1.1. Extreme Low Indoor Temperatures

The mean indoor air temperature of the 16 sample houses in Minginui, Te Whaiti and Murupara during the winter was only 11.8 °C, which is extremely low (see Table 2). The mean indoor air temperature of the 16 sample houses was lower than 18 °C for 65% of the time in autumn and 93% of the time in winter. For 46% and 86% of the time in autumn and winter and for 11 and 20.8 h per day, respectively, the mean indoor air temperature was lower than 16 °C. This study shows that for 16% and 55% of the time in autumn and winter and for 3.9 h and 13.2 h per day in autumn and winter, respectively, the mean indoor air temperature was lower than 12 °C. For 6% and 24% of the time in autumn and winter and for 1.4 h and 5.8 h per day in autumn and winter, respectively, the mean indoor air temperature was lower than 9 °C (see Table 3 and Table 4). The low indoor temperatures not only impact occupants’ thermal comfort but also occupants’ health conditions. According to the Respiratory Health Survey, 83% of participants from the sample houses in Minginui, Te Whaiti and Murupara had long-term physical or mental illness diagnosed by a doctor.

#### 3.1.2. High Indoor Relative Humidity

For 100% of the time with a range of 95% to 100% in autumn and winter, relative humidity adjacent to the floor (dust mites often grow in carpet) was equal to or higher than 50%: there would have been no limitations on dust mite survival and growth (see Table 3). For 96% (with a range of 73% to 100%) and 99% (with a range of 94% to 100%) of the time in autumn and winter, respectively, relative humidity adjacent to the floor was equal to or higher than 60%, which met the threshold of the reproduction of mites. For 51% (with a range of 0% to 100%) and 30% (with a range of 0% to 100%) of the time in autumn, mean relative humidity adjacent to the floor was equal to or higher than 75% and 80%, respectively. For 81% (with 2% to 100%) and 64% (with a range of 0% to 100%) of the time in winter, mean relative humidity adjacent to the floor was equal to or higher than 75% and 80%, respectively, which met the threshold of dust mites to thrive. Some sample houses were likely to have had a dust mite problem during the autumn and a worse situation during the winter.

On average there were only 21 days during the autumn (with a range of 0 to 87 days) when indoor relative humidity was equal to or higher than 80% for the 28 indoor spaces in the 16 sample houses (see Table 3), but on the test points adjacent to the ceilings in five indoor spaces and adjacent to the floors in nine indoor spaces, the time at this relative humidity was more than 30 days. On average there were only two days during the autumn (with a range of 0 to 36 days) when indoor relative humidity was equal to or higher than 90% for the 28 indoor spaces of the 16 sample houses, but on the test points adjacent to the ceilings in one indoor space and adjacent to the floors in two indoor spaces, the time at this relative humidity was more than seven days. According to the threshold for mould-spore germination conditions (see Table 1), mould spores could have germinated in some sample houses during the autumn. In addition, for 85% of the time in autumn (with range of 46% to 100%), indoor relative humidity was equal to or higher than 60%, which meets the threshold for mould growth conditions. This high indoor relative humidity could have caused early mould germination and mould problems during the autumn or the beginning of winter in some of the sample houses.

On average there were 42 days during the winter (with a range of 0 to 91 days) when indoor relative humidity was equal to or higher than 80% for the 25 indoor spaces of the 16 sample houses (see Table 3), which is clearly higher than the threshold for mould germination conditions. On the test points adjacent to the ceilings in seven indoor spaces and adjacent to the floors in 19 indoor spaces, the time at this relative humidity was more than 30 days. The time of mean indoor relative humidity for 14 indoor spaces was more than 30 days. In addition, for 90% of the time in winter (with a range of 51% to 100%), indoor relative humidity was equal to or higher than 60%, which meets the threshold for mould growth conditions. Most of the sample houses were likely to have had mould problems. According to the respiratory survey, 92% of participants from the sample houses in Minginui, Te Whaiti and Murupara reported mould problems on indoor surfaces.

### 3.2. Indoor Major Allergens

#### 3.2.1. Dust-Mite Allergens

According to the field study result of testing dust-mite allergens, all 13 sample houses in Minginui, Te Whaiti and Murupara had serious problems of indoor dust-mite allergens. There were seven samples with high levels of dust mite allergens and six sample houses with medium levels of dust-mite allergens. There were two sample houses with acceptable levels (the low level) of dust-mite allergens and one sample house without dust-mite allergen (undetectable dust-mite allergen) in Rotorua. Table 4 shows the mean indoor microclimatic conditions of the 15 sample houses with different levels (low, medium, and high levels) of dust-mite allergens.

Based on a comparison of indoor microclimatic conditions between the houses with acceptable levels (the low level) of dust-mite allergens and the houses with high and medium levels of dust-mite allergens, the indoor mean relative humidity adjacent to the floor (69.8%) of the houses with acceptable levels of dust-mite allergens was lower than 70% and clearly lower than 75%, the threshold for dust mites to thrive; the indoor mean relative humidity adjacent to the floor of the houses with high allergen levels (78%) and medium allergen levels (87.4%) was clearly higher than 75%.

For the houses with high and medium dust-mite allergen levels, the indoor relative humidity met the threshold for dust mites to thrive; the main difference was the indoor mean air temperature. The indoor mean air temperature (13.1 °C) of the houses with high allergens was 3.5 °C higher than that of the houses with medium allergens (9.6 °C). The mean air temperature adjacent to the floor (11.2 °C) of the houses with high allergens was 3.2 °C higher than the houses with medium allergens (8.0 °C). Dust mites prefer warmer and humid conditions for growth. If temporary space heating in a house without sufficient insulation cannot increase indoor temperature to the level that can decrease and control the indoor relative humidity to below the threshold for dust mites to thrive, a limited increase in the indoor mean air temperature (a couple of degrees increase from a very low baseline) can increase indoor dust-mite allergens, which can make indoor health conditions worse.

According to field-study data of two Rotorua houses, to control indoor dust-mite allergens at an acceptable level, indoor mean relative humidity adjacent to the floor must be maintained below 70%, and indoor relative humidity adjacent to the floor must be maintained below 75% (the threshold for dust mites to thrive) for 20 h (19.7 h in Table 4) a day during winter and below 80% all the time (99% of winter time or 23.8 h per day in Table 4) in winter. To achieve these conditions, the minimum indoor mean temperature must be maintained at 17 °C (16.7 °C in Table 4) or higher. The percentage of winter time in the two Rotorua houses, when indoor mean relative humidity was equal to or more than 80%, was only 1% (see Table 4), or 9.2 winter days, which was much lower than the threshold (30 days) for mould germination (Table 1). If the mould spores never germinate in a house, mould will never grow on indoor surfaces. If indoor dust-mite allergens can be controlled at the acceptable level, the house is unlikely to have a mould problem under the local climate. For a temperate climate with mild and wet winter, the threshold of indoor air temperature and relative humidity for the acceptable level of dust-mite allergens can be used as the minimum requirement for indoor thermal conditions to control indoor allergens at acceptable levels.

#### 3.2.2. Mould

In total, 16 sample houses were used for the field study of testing mould. In all three sample houses in Rotoura, there were no mould or mould spores detected. In all 13 sample houses houses in Minginui, Te Whaiti and Murupara, some mould or mould spores were detected on the indoor test areas. *Stachybotrys* was not detected in any sample houses. Test results show that *Cladosporium* was the only identified type of mould and had the highest frequency of detected mould in the 13 sample houses. *Cladosporium* is a well-known trigger for asthma [35,36,37]. An abundant level of *Cladosporium* on the five indoor surfaces of three sample houses, a moderate level of *Cladosporium* on the five indoor surfaces of four sample houses, and a low level of *Cladosporium* on the indoor surfaces of one sample house were identified. There was no *Cladosporium* detected on the indoor surfaces of five sample houses. A moderate level of unidentified fungus on the indoor surfaces of five sample houses and a low level of unidentified fungus on the indoor surfaces of two sample houses were noted. Mould test results can be influenced by occupants’ daily life and depend on how often the occupants clean the indoor surfaces, especially areas with visual mould.

Table 5 shows winter indoor mean microclimatic conditions and different *Cladosporium* growth levels of the 13 sample houses in Minginui, Te Whaiti and Murupara. For most of the winter time (over 80%), the indoor mean relative humidity of the houses with an abundant level or a moderate level of *Cladosporium* was higher than 60%, which meets the threshold of mould survival and growth (see Table 5). The time in winter, in the houses with abundant (33 days) and moderate (38 days) levels of *Cladosporium*, when indoor mean relative humidity was equal to or above 80%, was clearly higher than the threshold for mould germination (30 days). The main difference was the indoor mean temperature. Indoor mean temperatures in the houses with abundant levels of *Cladosporium* were 1 °C higher than in the houses with moderate levels of *Cladosporium*. Mould also prefers warm and humid conditions for growth. If temporary space heating in a house without sufficient insulation cannot increase indoor temperature to a level that can decrease and control indoor relative humidity to below the threshold for mould germination, a limited increase of indoor air temperature can create a better thermal condition for mould growth. Table 5 shows that the conditions of relative humidity and temperature were similar in houses that showed no or low levels of *Cladosporium* and houses with abundant or moderate levels of *Cladosporium*. This does not necessarily mean there could not have been higher levels of *Cladosporium* in these houses, but that it was present at low levels at the time of testing. This could be explained by cleaning, by the location of sampling, or other factors. In total, 92% of participants from the sample houses in Minginui, Te Whaiti and Murupara in the Respiratory Health Survey reported mould problems on indoor surfaces.

#### 3.2.3. Correlation between Dust Mites and Mould

According to the field study results of testing dust-mite allergens and mould growth in the 13 sample houses in in Minginui, Te Whaiti and Murupara (see Table 6), most of the sample houses with a high level of dust-mite allergens also had an abundant or moderate level of *Cladosporium*, and most of the sample houses with a medium level of dust-mite allergens also had low to moderate levels of *Cladosporium*. During most of the time in winter, the indoor mean relative humidity of the sample houses was higher than 50% and 60% (thresholds of dust-mite survival and mould growth conditions, respectively). The threshold (75–80% or higher) of relative humidity for dust mites to thrive and the threshold (80% or higher) of relative humidity for mould spore germination are quite close. If a house has high or medium levels of dust-mite allergens, it is likely to have a mould growth problem, and vice versa. The occupants can find visible mould growth on indoor surfaces but cannot see dust mites in the carpet.

### 3.3. Respiratory Health Survey

Table 7 shows the partial results of the Respiratory Health Survey. Group 1 in Table 7 includes all participants (20 occupants) from 15 sample houses in Minginui, Te Whaiti, Murupara and Rotorua; a very high percentage of participants in group 1 had respiratory symptoms. Over 40% of participants had wheezing or whistling symptoms, over 40% of participants had shortness of breath symptoms, over 40% of participants had chronic cough problem during the winter, over 35% of participants usually brought up sputum when they coughed, 10% of participants visited a hospital emergency department with breathing problems and over 25% of participants had sleeping problem related to their respiratory symptoms. In total, 20% of participants had asthma, which is significantly higher than the average (12.5%) of New Zealand. In total, 55% of participants had long-term physical or mental illness diagnosed by a doctor. The majority of sample houses where the participants came from are old houses with poor indoor thermal conditions (see Table 2 and Table 3) in Minginui, Te Whaiti and Murupara.

Based on results of the Respiratory Health Survey, the field study results of testing dust-mite allergens and mould, and field study results of indoor microclimate, group 2 in Table 7 includes the participants (12 occupants) from the 12 sample houses sample houses in Minginui, Te Whaiti and Murupara, which had high or medium levels of dust-mite allergens and abundant or moderate levels of mould growth (*Cladosporium* or unidentified fungus). The extremely high percentage of participants in group 2 had respiratory symptoms. Over 67% of participants had wheezing or whistling symptoms (this result is about twice as high as the prevalence in other studies [38]), over 50% of participants had shortness of breath symptoms, over 67% of participants had chronic cough problem during the winter, over 58% of participants usually brought up sputum when they coughed, 17% of participants visited a hospital emergency department with breathing problems and over 42% of participants had sleeping problems related to their respiratory symptoms. In total, 33% of participants had asthma, which is 2.6 times higher than the average (12.5%) of New Zealand. In total, 83% of participants had long-term physical or mental illness diagnosed by a doctor. The extremely high percentage of participants in group 2 who had respiratory symptoms is strongly associated with the high or medium levels of indoor dust-mite allergens and abundant or moderate mould of the sample houses in the Minginui, Te Whaiti and Murupara areas. Those old sample houses with poor thermal performance not only had unhealthy indoor thermal conditions but also had high indoor relative humidity during autumn and winter, which can cause serious indoor allergen problems from dust-mite and mould. Those indoor allergens and unhealthy thermal conditions can negatively impact occupants’ health conditions.

Group 3 in Table 7 includes the participants (eight occupants) from the three sample houses with basic or sufficient insulation and adequate space heating in Rotorua; the low percentage of participants in group 2 who had respiratory symptoms is apparently associated with the low levels of indoor dust-mite allergens or no dust-mite allergens detected and no mould in the three houses in Rotorua. For the local houses designed for temporary space heating, sufficient insulation in the building envelope and double-glazing, in compliance with the current building codes, are crucial to maintain indoor health conditions. The sufficient insulation in the building envelope and double-glazing could raise the baseline winter indoor temperatures and significantly reduce indoor mean relative humidity; it would then be possible in those houses with adequate temporary space heating to maintain the indoor relative humidity below the threshold for mould to germinate and dust mites to thrive.

## 4. Discussion

Based on field-study data of indoor microclimates, dust-mite allergens and mould growth, and occupants’ Respiratory Health Survey data from the sample houses with insufficient insulation and poor thermal performance, the study provided evidence and physical data to prove that poor health conditions of occupants are closely related to their poor indoor living conditions. Houses with poor thermal performance and insufficient insulation can have extremely low indoor temperatures and serious indoor allergens from dust mites and mould, which can directly harm or negatively impact occupants’ health.

The study identified that not only in winter, but also in autumn, the indoor relative humidity of the sample houses with insufficient insulation met the threshold conditions for mould to germinate and dust mites to thrive. Some sample houses were likely to have had dust mite and mould problems during the autumn and a worse situation during the winter. Occupants could suffer from dust-mite and mould allergies for a long period of time every year. The study identified strong correlations between dust-mite and mould problems in indoor spaces of the sample houses with insufficient insulation. If a house had a dust-mite allergen problem (medium or high levels of dust-mite allergens), it was likely to have a mould problem, and vice versa, in a temperate climate with mild and wet winters.

Dust mites and mould prefer warm and humid conditions. If temporary space heating in a house with insufficient insulation cannot increase the indoor temperature to a level that can decrease and maintain the indoor relative humidity below the threshold for mould to germinate and dust mites to thrive, the limited increase in indoor air temperature (a couple of degrees increase from a very low baseline) can create a better thermal condition for dust mite development and mould growth, and indoor health conditions can become worse.

For a local conventional house with lightweight timber structure, designed for temporary heating, under a temperate climate with a mild and wet winter, it is possible for a house (a retrofitting old house or a new house) with sufficient insulation in its building envelope and temporary space heating to maintain indoor health living conditions. Sufficient insulation and double-glazed windows could raise the baseline winter indoor temperatures and significantly reduce indoor mean relative humidity; it would then be possible in those houses with adequate temporary space heating to maintain the indoor relative humidity below the threshold for mould to germinate and dust mites to thrive. If there were no mould spore germination, there would be no problem of mould growth on indoor surfaces. If dust-mite allergens were controlled at a low or undetectable level, there would be no dust-mite allergy problems in indoor spaces.

This is the first state-funded, cross-disciplinary collaborative research project studying the health and living conditions of an isolated indigenous community in remote areas of New Zealand. The study provides first-hand field study data and physical evidence to identify that the poor health of occupants of the isolated Māori communities is closely related to their inadequate living conditions, which can attract the government and health authority’s attention to occupants’ health conditions and their living conditions in remote and economically disadvantaged regions of New Zealand. A new housing project with 33 new houses, funded by the Ministry of Māori Development in 2020, is going to be developed by Matekuare Whanau Trust for the local Māori residents at the same study site (Te Whaiti). The new housing development can potentially become a national sample of affordable, healthy and sustainable housing development for the isolated Māori communities. The new houses can be used for a further comparison study of occupants’ health conditions with different living conditions of the local Māori community.

The sites for the field studies and the Respiratory Health Survey for the isolated Māori communities are in the remote areas with a very low population density. Maori occupants in those communities have very large variation of residential mobility in different families due to many different reasons. This residential mobility may affect the duration of living in dwellings. It is difficult for the Respiratory Health Survey to have a large number of participants who are the owners of the sample houses or the long-term tenants of the sample houses. Although the sample size of the respiratory survey results was small, a strong correlation between the respiratory survey results and dust mite and mould test results can be still identified. The extremely high percentages of occupants who had respiratory symptoms were strongly associated with the high levels of indoor dust mites and mould allergens in the sample houses in the Minginui, Te Whaiti and Murupara areas. The low percentages of participants who had respiratory symptoms were apparently associated with the low levels of indoor dust mites and mould allergens in the sample houses in Rotorua.

## 5. Conclusions

According to field-study data, to control indoor dust-mite allergens at a low (acceptable) level, indoor mean relative humidity adjacent to the floor must be maintained below 70%, and indoor relative humidity adjacent to the floor must be maintained below 75% (the threshold for dust mites to thrive) for 20 h a day during winter and below 80% all the time in winter. To achieve these conditions, the indoor mean temperature must be maintained at 17 °C or higher. If indoor relative humidity adjacent to the floor can be controlled below the threshold for dust mites to thrive (75%) for 20 h a day during the winter, it will not reach the threshold for mould germination. If the mould spores never germinate in a house, mould will never grow on indoor surfaces. If indoor dust-mite allergen levels can be controlled at low (acceptable) levels, the house is unlikely to have a mould problem in a temperate climate with mild and wet winters. If indoor relative humidity adjacent to the floor can be controlled below the threshold for dust mites to thrive during the winter, indoor dust-mite allergens can be controlled at an acceptable level and mould growth on indoor surfaces can be prevented, which can be used as a guideline or strategy for new house development or old house retrofitting, with adequate space heating methods, to minimise indoor allergy problems and maintain healthy indoor conditions for occupants under the local climate with a mild and wet winter.

## Figures and Tables

**Table 1:** The thresholds of relative humidity (RH) and time for mould germination [30]

Substrate	Threshold of RH	Time Needed
Porous and dust- and fat-covered non-porous	100%	1 day
	89%	7 days
	80%	30 days

Source: H.L.S.C. Hens, Minimising Fungal Defacement.

**Table 2:** Autumn and winter time related to different ranges of mean indoor temperatures of the 16 sample houses in Minginui, Te Whaiti and Murupara.

Seasons	Indoor Mean Temperature	Temperature Ranges	% of Time (Range) (%)	Time Per Day (Range) (Hour)	Outdoor (Day)
Autumn	16.5 °C	T < 9 °C	6 (0–25)	1.4 (0–6.0)	17.1
		T < 10 °C	8 (0–32)	2 (0–7.7)	22.6
		T < 12 °C	16 (0–52)	3.9 (0–12.5)	35.5
		T < 14 °C	29 (2–72)	6.8 (0.5–17.3)	50.5
		T < 16 °C	46 (10–88)	11 (2.4–21.1)	67.1
		T < 18 °C	65 (26–96)	15.5 (6.2–23)	79.9
Winter	11.8 °C	T < 9 °C	24 (0–67)	5.8 (0–16.1)	49.2
		T < 10 °C	34 (0–81)	8.1 (0–19.4)	59.1
		T < 12 °C	55 (3–96)	13.2 (0.7–23)	76.9
		T < 14 °C	74 (22–100)	17.7 (5.3–24)	87.9
		T < 16 °C	86 (55–100)	20.8 (13.2–24)	91.4
		T < 18 °C	93 (68–100)	22.4 (16.3–24)	91.8

**Table 3:** Autumn and winter time related to different ranges of mean indoor relative humidity of the 16 sample houses in Minginui, Te Whaiti and Murupara.

Spaces	Indoor Mean (Range)		Ceiling Mean (Range)		Floor Mean (Range)		Outdoor
Autumn	% of time (%)	Time (Day)	% of time (%)	Time (day)	% of Time (%)	Time (day)	% of Time (%)
RH ≥ 50%	95 (73–100)	87 (66–92)	91 (46–100)	84 (42–92)	100 (95–100)	92 (87–92)	99
RH ≥ 60%	85 (46–100)	78 (42–92)	74 (19–100)	68 (18–92)	96 (73–100)	88 (67–92)	96
RH ≥ 75%	40 (0–98)	37 (0–90)	28 (0–97)	26 (0–89)	51 (0–100)	47 (0–92)	70
RH ≥ 80%	22 (0–94)	21 (0–87)	15 (0–89)	14 (0–82)	30 (0–100)	27 (0–92)	57
RH ≥ 90%	3 (0–40)	2 (0–36)	2 (0–30)	2 (0–28)	4 (0–49)	3 (0–45)	22
Winter	% of time (%)	Time (day)	% of time (%)	Time (day)	% of time (%)	Time (day)	% of time (%)
RH ≥ 50%	96 (63–100)	88 (58–92)	92 (27–100)	85 (25–92)	100 (98–100)	92 (90–92)	100
RH ≥ 60%	90 (51–100)	83 (47–92)	81 (5–100)	75 (5–92)	99 (94–100)	91 (86–92)	98
RH ≥ 75%	65 (1–100)	56 (1–91)	49 (0–100)	42 (0–91)	81 (2–100)	71 (2–91)	82
RH ≥ 80%	48 (0–100)	42 (0–91)	31 (0–100)	25 (0–91)	64 (0–100)	54 (0–91)	70
RH ≥ 90%	10 (0–83)	8 (0–75)	6 (0–77)	5 (0–70)	13 (0–89)	9 (0–81)	35

**Table 4:** Winter indoor mean microclimatic conditions of the sample houses with different levels of dust-mite allergens.

Dust-Mite Allergen	High (7 Houses)		Medium (6 Houses)		Low (2 Rotorua Houses)		
Test Points	Indoor Mean	Floor Mean	Indoor Mean	Floor Mean	Indoor Mean	Floor Mean	
Mean T	13.1 °C	11.2 °C	9.6 °C	8.0 °C	16.7 °C	15.2 °C	
Mean RH	71.2%	78.0%	80.8%	87.4%	63.9%	69.8%	
	**Percentage of Time in Winter**						**Time/Day**
T ≥ 16 °C	21%	4%	3%	0%	60%	35%	8.4 h/d
T ≥ 18 °C	12%	0%	1%	0%	34%	5%	4.3 h/d
T ≥ 20 °C	7%	0%	1%	0%	9%	0%	1.2 h/d
RH ≥ 70%	63%	80%	86%	96%	19%	51%	12.2 h/d
RH ≥ 75%	49%	65%	76%	93%	4%	18%	4.3 h/d
RH ≥ 80%	30%	45%	61%	88%	0%	1%	0.2 h/d

**Table 5:** Winter indoor microclimatic conditions of the 13 sample houses with different growth levels of *Cladosporium*.

*Cladosporium*	Abundant (3 Houses)		Moderate (4 Houses)		None or Low (6 Houses)	
Mean T	12.4 °C		11.4 °C		11.5 °C	
Mean RH	72.3%		74.5%		75.9%	
**Winter Time**	**% of Time**	**Time**	**% of Time**	**Time**	**% of Time**	**Time**
T ≥ 16 °C	20%	18day	13%	12 day	11%	10 day
T ≥ 18 °C	13%	12day	9%	8 day	5%	5 day
T ≥ 20 °C	8%	7day	6%	6 day	2%	2 day
RH ≥ 60%	80%	74day	87%	80 day	90%	83 day
RH ≥ 75%	52%	47day	62%	57 day	60%	55 day
RH ≥ 80%	36%	33day	42%	38 day	41%	38 day

**Table 6:** Sample houses with high or medium levels of dust-mite allergens and mould in test results.

Sample Houses	Dust-Mite Allergens	*Cladosporium*	Unidentified Fungus
Sample house 1	High	Moderate	-
Sample house 2	High	Abundant	-
Sample house 3	High	-	Low
Sample house 4	High	Abundant	-
Sample house 5	High	-	-
Sample house 7	High	Abundant	-
Sample house 6	High	-	Low to Moderate
Sample house 8	Medium	Moderate	Low
Sample house 9	Medium	-	Moderate
Sample house 10	Medium	Low	Low to moderate
Sample house 11	Medium	-	Moderate to abundant
Sample house 12	Medium	Moderate	Moderate to abundant
Sample house 13	Medium	Low to moderate	Low

**Table 7:** Partial Respiratory Health Survey results of the three groups of participants.

Major Questions for Respiratory Symptoms	Group 1	Group 2	Group 3
Had wheezing or whistling in the last 12 months	45%	75%	0%
Had breathless when the wheezing noise was present	40%	67%	0%
Had wheezing or whistling when you did not have a cold	40%	67%	0%
Had a daytime attack of shortness of breath during rest time	40%	50%	25%
Had an attack of shortness of breath caused by physical activity	60%	83%	25%
Visited doctor for breathing problems or shortness of breath	60%	83%	25%
Usually coughed first thing in the morning in winter	45%	75%	0%
Usually coughed during the day or night in winter	50%	67%	25%
Coughed during the day or night for three months a year	40%	67%	0%
Usually brought up sputum first thing in winter morning	35%	58%	0%
Usually brought up sputum during winter day or night	45%	67%	13%
Brought up sputum during the day or night for 3 months a year	35%	58%	0%
Had trouble with your breathing ever before	30%	50%	0%
Visited emergency department because of breathing problems	10%	17%	0%
Had asthma	20%	33%	0%
Woken up with chest tightness in the last 12 months	25%	42%	0%
Woken by an attack of coughing in the last 12 months	60%	75%	38%
Woken up at night and had trouble falling back to sleep	55%	67%	38%
Had any mould or mildew on any indoor surface	70%	92%	38%
Had any nasal allergies	40%	50%	25%
Had eczema or any kind of skin allergy	60%	75%	38%
Had long-term physical or mental illness diagnosed by doctor	55%	83%	13%

## References

[1] WHO (2006). WHO Second Technical Meeting on Quantifying Disease from Inadequate Housing.

[2] D’Amato M., Molino A., Calabrese G., Cecchi L., Annesi-Maesano I., D’Amato G. (2018). The impact of cold on the respiratory tract and its consequences to respiratory health. Clin. Transl. Allergy.

[3] WHO (2011). Environmental Burden of Disease Associated with Inadequate Housing: A Method Guide to the Quantification of Health Effects of Selected Housing Risks in the WHO European Region.

[4] Collins K.J. (1986). Low indoor temperatures and morbidity in the elderly. Age Ageing.

[5] Barnard L.T., Zhang J. (2018). The Impact of Respiratory Disease in New Zealand: 2018 Update.

[6] Ministry of Health (2018). New Zealand Health Survey 2017/18.

[7] Nielsen K.G., Bisgaard H. (2005). Hyperventilation with cold versus dry air in 2- to 5-year-old children with asthma. Am. J. Respir. Crit. Care Med..

[8] WHO (1987). Health Impact of Low Indoor Temperatures: Report on a WHO Meeting: Copenhagen, 11–14 November 1985.

[9] WHO (2009). WHO Guidelines for Indoor Air Quality: Dampness and Mould.

[10] Bailie R.S., Wayte K.J. (2006). Housing and health in Indigenous communities: Key issues for housing and health improvements in remote Aboriginal and Torres Strait Islander Communities. Aust. J. Rural Health.

[11] McCartney S., Herskovits J., Trnavsky K. Indigenous Housing: Towards a Model Supporting Community Health. https://www.alternativesjournal.ca/sustainable-living/indigenous-housing-towards-model-supporting-community-health.

[12] Lloyd E.L. (1990). Hypothesis: Temperature recommendations for elderly people: Are we wrong?. Age Ageing.

[13] Hunt S., Charlton J., Murphy M. (1997). Housing-related disorders. The Health of Adult Britain.

[14] Goodwin J., Nicol F., Rudge J. (2000). Cold stress, circulatory illness and the elderly. Cutting the Cost of Cold: Affordable Warmth for Healthier Homes.

[15] Arundel A.V., Sterling E.M., Biggin J.H., Sterling T.D. (1986). Indirect health effects of relative humidity in indoor environment. Environ. Health Perspect..

[16] Su B., Schnabel M.A. (2017). Field study of Auckland housing winter indoor health conditions associated with insulation, heating and energy. Proceedings of the 51st International Conference of the Architectural Science Association (ANZAScA).

[17] Arlian L.G., Bernstein I.L., Gallagher J.S. (1982). The prevalence of house dust mites, *Dermatophagoides* spp, and associated environmental conditions in homes in Ohio. J. Allergy Clin. Immunol..

[18] Korsgaard J. (1982). Preventive measures in house-dust allergy. Am. Rev. Respir. Dis..

[19] Murray A.B., Zuk P. (1979). The seasonal variation in a population of house dust mites in a North American city. J. Allergy Clin. Immunol..

[20] Arlian L.G., Yella L., Morgan M.S. (2010). House dust mite population growth and allergen production in cultures maintained at different temperatures. J. Allergy Clin. Immunol..

[21] Arlian L.G., Neal J.S., Vyszenski-Moher D.L. (1999). Reducing relative humidity to control the house dust mite *Dermatophagoides farinae*. J. Allergy Clin. Immunol..

[22] Arlian L.G., Rapp C.M., Ahmed S.G. (1990). Development of Dermatophagoides pteronyssinus (Acari: Pyroglyphidae). J. Med. Entomol..

[23] Hart B.J. (1998). Life cycle and reproduction of house-dust mites: Environmental factors influencing mite populations. Allergy.

[24] American Society of Heating, Refrigeration and Air-Conditioning (ASHRAE) (2000). ASHRAE Standard 62-2000—Ventilation for Acceptable Indoor Air Quality.

[25] Department of Building and Housing (2001). Compliance Document for New Zealand Building Code—Clause G5 Interior Environment.

[26] Standards New Zealand (1990). New Zealand Standard 4303:1990 Ventilation for Acceptable Indoor Air Quality.

[27] Coppock J.B.M., Cookson E.D. (1951). The effect of humidity on mould growth on constructional materials. J. Sci. Food Agric..

[28] Block S.S. (1953). Humidity requirements for mould growth. Appl. Microbiol..

[29] Pasanen A.L., Juutinen T., Jantunen M.J., Kalliokoski P. (1992). Occurrence and moisture requirements of microbial growth in building materials. Int. Biodeterior. Biodegrad..

[30] Hens H.L.S.C. (2000). Minimising fungal defacement. Ashrae J..

[31] Su B. (2006). Prevention of winter mould growth in housing. Archit. Sci. Rev..

[32] American Society of Heating, Refrigeration and Air-Conditioning (ASHRAE) (1993). Thermal insulation and vapour retarders applications. ASHRAE Handbook—Fundamentals.

[33] Irwin G., Walrond C. (2005). When Was New Zealand First Settled? Te Ara—The Encyclopedia of New Zealand. http://www.teara.govt.nz/en/when-was-new-zealand-first-settled.

[34] Janson C., Anto J., Burney P., Chinn S., de Marco R., Heinrich J., Jarvis D., Kuenzli N., Leynaert B., Luczynska C. (2001). The European Community Respiratory Health Survey: What are the main results so far?. Eur. Respir. J..

[35] Peternel R., Culig J., Hrga I. (2004). Atmospheric concentrations of *Cladosporium* spp. and *Alternaria* spp. spores in Zagreb (Croatia) and effects of some meteorological factors. Ann. Agric. Environ. Med..

[36] Flannigan B., Samson R.A., Miller J.D. (2001). Microorganisms in Home and Indoor Work Environments: Diversity, Health Impacts, Investigation and Control: Abingdon-on-Thames.

[37] Piecková E., Jesenská Z. (1999). Microscopic fungi in dwellings and their health implications in humans. Ann. Agric. Environ. Med..

[38] Pescatore A.M., Spycher B.D., Beardsmore C.S., Kuehni C.E. (2015). “Attacks” or “Whistling”: Impact of questionnaire wording on wheeze prevalence estimates. PLoS ONE.

